# Fever as a first presentation of castration-resistant prostate cancer: A case report

**DOI:** 10.1097/MD.0000000000029428

**Published:** 2022-07-29

**Authors:** Tae Hoon Oh, Seung Chol Park

**Affiliations:** Department of Urology, Wonkwang University Hospital, Institute of Wonkwang Medical Science, Iksan, Korea.

**Keywords:** fever, prostate cancer

## Abstract

**Rationale:**

Cancer is a well-recognized cause of fever, which is related to cytokines produced by malignant cells. Prostate cancer presenting with fever and other inflammatory markers as a paraneoplastic syndrome rarely occurs.

**Patients concerns and diagnoses:**

We describe the case of high fever and lower-urinary tract symptoms that progressed 1 month prior to presentation. A 78-year-old man had been diagnosed with prostate cancer 8 months ago. He received androgen deprivation therapy with leuprolide acetate 22.5 mg for every 3 months. Castration-resistant prostate cancer was diagnosed due to elevated prostate specific antigen (1639 ng/mL) and cancer fever.

**Intervention:**

The patient received docetaxel-based systemic chemotherapy 50 mg/mm^2^ biweekly. Naproxen 500 mg was administered twice a day.

**Outcomes:**

After one cycle of systemic chemotherapy, the patient had no major side effects, no more fever was observed, and the systemic condition improved.

**Conclusion:**

Differentiating cancer-related fever from infection-related fever is important for appropriate patient management. In this case, fever appeared as the first symptom of castration-resistant prostate cancer and was managed by naproxen and resolved with systemic chemotherapy.

## 1. Introduction

Fever is an important host-defense response accompanied by inflammation. It is one of the most common symptoms and signs of outpatients and inpatients. The definition of fever is arbitrary and depends on the purpose for which it is defined. In patients with neutropenia, fever is defined as a single oral temperature of >38.3°C.^[1]^ Cancer can cause fever. The pathophysiology of cancer-induced fever maybe a result of several mechanisms, including release of cytokines from tumor cells, necrosis of tumoral tissue, or obstruction of a hollow duct. Other causes of fever in cancer patients include drug-induced fever (antibiotics or chemotherapeutic agents), thrombotic thrombocytopenic purpura, and deep venous thrombus.^[2]^ Cancer-induced fever is associated with lymphoma, leukemia, and solid tumors. Solid tumors that result in cancer-induced fever include renal cell carcinoma, hepatocellular carcinoma, pancreatic carcinoma, and brain tumor.^[3]^

Castration-resistant prostate cancer (CRPC) is defined as castrated serum testosterone <50 ng/dL, plus one of following biochemical radiological progressions.^[4,5]^ Primary symptoms of CRPC are bone pain and skeletal events. Fever and inflammatory paraneoplastic syndromes associated with CRPC are extremely rare.

## 2. Case presentation

A 78-year-old Korean man was admitted with high fever (up to 38°C), poor feeding, knee pain, and lower-urinary tract symptoms that progressed over the preceding month. He had been diagnosed with prostate cancer ~8 months ago. At initial admission, the serum prostate specific antigen (PSA) level was over 100 ng/mL and prostate volume was 84 ml. The biopsy result showed Gleason grade was 1 in all cores. A computed tomography showed bladder and seminal vesicle invasion of prostate cancer, multiple enlarged pelvic lymph nodes, and osteoblastic bone metastases in spines, pelvic bones, and both proximal femurs. He was treated with androgen deprivation therapy with leuprolide acetate 22.5 mg every 3 months. He was treated well and had only mild lower urinary tract symptoms. Imaging tests performed 3 months prior to visit showed a stable disease, the serum PSA levels decreased to 13 ng/mL.

**Figure 1.**
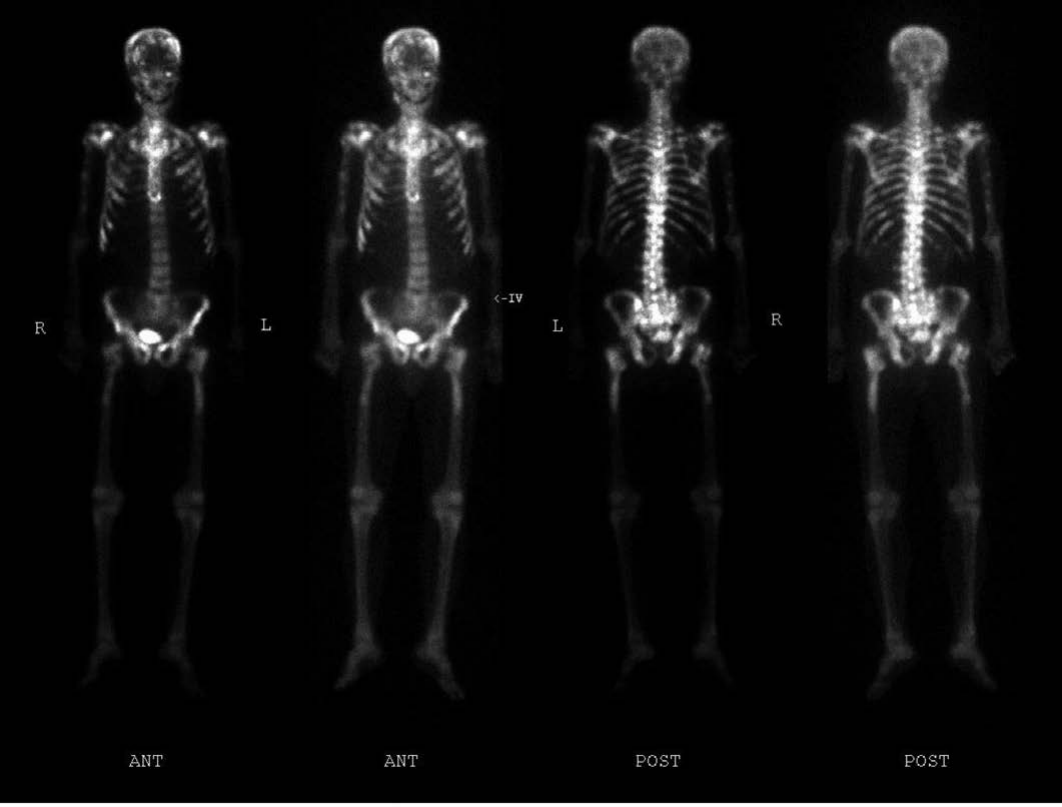
Bone scintigraph showing extensive bone metastases.

On physical examination, he had a regular peripheral pulse of 101 bpm and a blood pressure of 140/70 mm Hg. He was febrile with a body temperature of 38.8°C. Laboratory test revealed the following results: hemoglobin 12.8 g/dL; WBC 11,980/μl; platelet 147,000/μL; C-reactive protein 114 mg/dL; serum PSA 1639 ng/mL. Urine analysis revealed no hematuria and pyuria. No bacteria had grown in the urine culture test and blood culture test. COVID-19 test was negative. Serum procalcitonin level had increased to 0.61 ng/mL (<0.1 ng/mL) and so did the serum interleukin 6 level increase to 307 pg/mL (<7.0 pg/mL). According to the opinion of the Infectious Internal Medicine, third-generation intravenous cephalosporin and nonsteroidal anti-inflammatory drugs were administered, but the fever persisted for more than 3 days. Naproxen 500 mg was administered twice a day and the fever improved to a certain extent. No new lesion was observed on the imaging test (Fig. 1), but CRPC was diagnosed due to elevated PSA and cancer fever. Hence, docetaxel-based systemic chemotherapy (50 mg/m^2^ biweekly) was initiated. After 1 cycle of systemic chemotherapy, the patient had no major side effects, no more fever was observed, and the systemic condition improved. The patient received 22 cycles of systemic docetaxel chemotherapy during the 14-month follow-up and still suffered from a stable disease. The last serum PSA level was 10 ng/mL. This study was approved by Wonkwang University Hospital Institutional Review Board (WKUH 2021-05-018). Patient has provided informed consent for publication for the case.

## 3. Discussion

In cancer patients, fever is a common symptom for various reasons. In certain cancers, cancer itself causes fever, and typical cancers that cause fever are lymphoma, leukemia, and solid tumor. Some specific solid malignancies that result in cancer fever include renal cell carcinoma (by elaboration of interleukin-6), hepatocellular carcinoma, pancreatic carcinoma, bronchogenic carcinoma, and brain tumor.^[3]^ Patients with cancer are exposed to a variety of treatment situations, blood products, and medications that may induce fever. Infection is a major cause for it in cancer patients. Risk factors of fever in cancer patients include venous catheters, chemotherapy-induced mucositis, various surgical procedures, and foreign bodies.^[5]^ Deep vein thromboembolism is an important diagnosis that may occur in patients with cancer and should be considered in any patient without clear evidence of infection-associated fever. Diagnostic criteria for cancer fever included a temperature of over 37.8°C at least once each day, lasting for over 2 weeks, a lack of evidence of infection, absence of alleged mechanisms, that is, drug allergy, transfusion reaction, lack of response of fever to an empiric, adequate antibiotics, prompt, complete lysis of fever by the naproxen test.

In any cancer patient presenting with fever, it is very important to distinguish cancer from an infection. Fever can occur when acute prostatitis develops in patients with prostate cancer. Clinically, when acute prostatitis occurs, PSA increases by an average of 20 ng/mL.^[6]^ In this case, PSA increased to 1000 ng/mL or more, and infection could be ruled out because bacteria were not cultured in urine culture tests and blood culture tests. Procalcitonin is an important marker to determine the severity of bacteremia or sepsis. Yaegashi et al suggested that procalcitonin was significantly higher in bacterial infections in advanced urological cancer, which was useful for differential diagnosis.^[7]^ In this case, procalcitonin was slightly increased to 0.61 ng/mL, indicating that it was not an infection-induced fever.

Across various solid tumors, fever is caused by several cytokines such as interleukin-2, TNF, and interleukin-6. In particular, interleukin-6 is involved in fever associated with renal cell carcinoma. Inflammatory cytokines are known to play an important role in the progression and prognosis of prostate cancer. Mauri et al report an association between high serum levels of interleukin-6, a pyrogenic molecule, and advanced prostate cancer.^[8]^ In this case as well, interleukin-6 increased to 307 pg/mL, which can be considered as a factor causing fever.

Prostate cancer presenting with fever and other inflammatory markers as a paraneoplastic syndrome is a rare occurrence. All patients with prostate cancer related systemic inflammation had metastatic prostate cancer. This finding suggests that systemic inflammation may be related with high tumor burden and a rapidly progressive course of prostate cancer. Fever and other related symptoms were resolved with androgen deprivation therapy in all cases reported.^[9]^ In this case, castration-resistant prostate cancer was diagnosed as a result of a sudden fever in a patient receiving androgen deprivation therapy. In this case, fever was controlled after administration of naproxen and resolved after systemic docetaxel chemotherapy. Although paraneoplastic syndrome is rare in prostate cancer, if fever persists unresponsive to broad spectrum antibiotics, rapid progression of the prostate cancer can be predicted and appropriate treatment decisions should be made.

## 4. Conclusions

Differentiating cancer-related fever from infection-related fever is important for appropriate patient management. In this case, fever appeared as the first symptom of castration-resistant prostate cancer. The fever was controlled by naproxen and resolved with systemic chemotherapy.

## Acknowledgments

This paper was supported by Wonkwang University in 2020.

We would like to thank Editage (www.editage.co.kr) for English language editing.

## Author contributions

Data curation; Tae Hoon Oh, Seung Chol Park

Writng - original draft: Tae Hoon Oh
